# A Non-Destructive Early Sex Identification Method for Chicken Embryos Based on Improved MobileViT-V3

**DOI:** 10.3390/ani16091377

**Published:** 2026-04-30

**Authors:** Qian Yan, Chengyu Yu, Zhoushi Tan, Zesheng Wang, Qiaohua Wang

**Affiliations:** 1 College of Engineering, Huazhong Agricultural University, Wuhan 430070, China; yqyq@webmail.hzau.edu.cn (Q.Y.); ycy010504@webmail.hzau.edu.cn (C.Y.); 2068409343@webmail.hzau.edu.cn (Z.T.); wangzesheng@webmail.hzau.edu.cn (Z.W.); 2 Ministry of Agriculture Key Laboratory of Agricultural Equipment in the Middle and Lower Reaches of the Yangtze River, Wuhan 430070, China; 3 National Research and Development Center for Egg Processing, Huazhong Agricultural University, Wuhan 430070, China

**Keywords:** attention mechanism, feature enhancement, hatching egg sex identification, MobileViT-V3, modern smart agriculture, non-destructive testing

## Abstract

The global poultry hatching industry faces severe challenges of resource waste and animal ethics issues due to the routine culling of day-old male chicks. Meanwhile, early sex identification of 4-day-incubated chicken embryos is limited by low accuracy, as embryos at this stage have weak, low-contrast blood vessels that are highly susceptible to interference from the eggshell’s texture. To address these issues, this paper proposes a non-destructive early sex identification method for chicken embryos based on an improved MobileViT-V3 model. Taking the lightweight hybrid architecture MobileViT-V3 as the backbone, we embedded a Micro Feature Enhancement module (MFE-Module) in Stage 3 to strengthen the extraction of fine vascular details, and a Multi-Scale Adaptive Attention Fusion module (MSAAF-Module) in Stage 4 to realize adaptive weighted screening of multi-source features. Experiments on the self-constructed dataset of 4-day-incubated embryos show that the improved model achieves a test set classification accuracy of 92.26%, with an F1-score of 92.15%, a recall rate of 92.12%, and a Kappa coefficient of 0.845. It outperforms mainstream models such as YOLOv12, ShuffleNetV2, ConvNeXt-T, ResNet, and Swin-ViT, with only 2.98 M parameters and an inference speed of 97.6 FPS, well exceeding the 30 FPS real-time requirement of industrial sorting lines and showing high potential for practical industrial deployment. This method provides a new scheme for non-destructive, high-precision, and high-efficiency early sex identification in poultry hatching.

## Simple Summary

Every year, around 7 billion newly hatched male chicks are culled globally in the egg-laying poultry industry, which causes huge resource waste and serious animal welfare issues. Early sex identification of chicken embryos before hatching can effectively solve this problem, but it is hard to identify the sex accurately at the early incubation stage due to the weak blood vessels of embryos. In this study, we developed a non-destructive, low-cost, and high-accuracy method for early sex identification of chicken embryos using an improved artificial intelligence model. This method achieved 92.26% identification accuracy on the fourth day of incubation, with a fast detection speed that meets the needs of industrial production. It can help reduce chick culling, improve animal welfare, and support the sustainable development of the poultry industry.

## 1. Introduction

China has long been the world’s largest producer in the poultry breeding and hatching egg industry, with an annual egg production exceeding 30 million tons for consecutive years, accounting for more than 35% of the global total [1]. The culling of day-old male chicks in layer breeding has long restricted the green development of the sector [2]. Layer-type roosters do not lay eggs, and their growth efficiency and meat performance are far lower than those of specialized broilers [3].

Approximately 7 billion male chicks are culled globally each year. This practice not only causes massive waste of hatching energy, labor, and space resources, but also goes against the core principles of animal welfare and sustainable industrial development [4]. Since Germany, France, and other countries legislated to ban the systematic culling of male chicks in 2022, early, non-destructive and high-throughput in ovo sex identification technology has become an urgent industrial demand [5]. Studies have confirmed that chicken embryos do not develop functional nociception until the second half of incubation (after Day 10), with no evidence of pain perception before Day 7 [6]. Sex identification and removal of male eggs on the fourth day of incubation can not only meet ethical requirements but also significantly reduce production costs. Therefore, the development of early non-destructive identification methods suitable for industrial assembly lines is of great practical significance.

Existing in ovo sex identification technologies have obvious shortcomings in industrial applications. Molecular biological methods such as qPCR [7] and allantoic fluid detection [8] achieve high accuracy, but they are invasive, costly, and slow, making them unsuitable for large-scale sorting. Spectroscopy [9,10,11], hyperspectral imaging [12,13,14], and other techniques can reach about 90% accuracy in the early and middle stages of egg incubation, but they rely on expensive equipment, have a low detection speed, and are susceptible to interference from the eggshell’s texture and illumination. Methods such as electronic nose [15] and magnetic resonance imaging [16] either require long detection cycles, involve high equipment costs, or pose biosafety risks, so they are difficult to popularize in hatcheries. Machine vision has become the most industrially promising technical route due to its non-contact, low-cost, and easy to deploy advantages.

However, the blood vessels of chicken embryos on the fourth day of incubation are weak, slender, low-contrast, and easily obscured by eggshell textures and illumination noise, representing a typical weak-target, fine-grained visual classification task. Traditional CNN models have limited receptive fields and struggle to capture long-range feature dependencies, resulting in limited robustness to interference for weak vascular features [17]. Native ViT lacks spatial inductive bias and shows insufficient perception of tiny vascular details [18]. Lightweight networks run fast but have limited feature expression, failing to meet industrial accuracy requirements. Thus, a deep learning model that balances global modeling, local fine-grained extraction, and lightweight deployment is urgently needed to achieve high-precision identification of weak blood vessels.

As a new-generation lightweight convolutional-Transformer hybrid architecture [19], MobileViT-V3 efficiently integrates the local spatial perception of CNN and the global modeling capability of Transformer. It performs stably in small-sample, weak-target and mobile scenarios, making it highly suitable for early in ovo sex identification. However, existing studies based on lightweight models still face two key gaps: (1) insufficient extraction capability for tiny, low-contrast vascular features in early-stage embryos; (2) a lack of adaptive feature screening mechanisms for complex interference in industrial scenarios. To address these limitations, this paper takes MobileViT-V3 as the backbone network and designs a Micro Feature Enhancement module and a Multi-Scale Adaptive Attention Fusion module to strengthen the extraction of weak vessel details and realize adaptive screening of multi-source features. Experiments on a self-constructed dataset of 4-day-old chicken embryos demonstrate that the improved model effectively enhances the robustness of weak features and classification accuracy. It can provide a lightweight, high-precision, and industrially deployable solution for non-destructive early sex identification of chicken embryos.

## 2. Materials and Methods

### 2.1. Experimental Materials and Instruments

This experiment was conducted in the modern hatchery of Shijiazhuang Yukou Poultry Industry Co., Ltd., Shijiazhuang City, Hebei Province, China. The hatching eggs used in this study were provided by Shijiazhuang Yukou Poultry Industry Co., Ltd., and were of the Jingfen No. 1 white-shell layer breed. This breed is a typical fast–slow feathering auto-sexing strain with stable genetic traits and high egg uniformity, making it well-suited for early sex identification of chicken embryos. A total of 4230 fresh hatching eggs with intact appearance, no cracks, no dirt, and a uniform eggshell texture were selected. The egg weight was controlled within the range of 50–60 g to reduce the interference of individual morphological differences on the experimental results. It should be noted that only Jingfen No. 1 white-shell layer eggs were used in this study, which may limit the generalizability of the model to other layer breeds or eggshell colors (e.g., brown-shell or pink-shell eggs).

Before incubation, the surfaces of hatching eggs were wiped and disinfected, air-dried naturally, and then kept at 20 °C for 24 h to stabilize the embryonic development environment. Subsequently, the eggs were incubated in an internationally advanced microcomputer-controlled fully automatic incubator. This equipment is equipped with automatic temperature control, humidity regulation, egg turning, and ventilation functions, which can accurately simulate the natural incubation environment to ensure the optimal embryonic development of the hatching eggs.

To minimize the influence of individual differences in embryonic development on the identification results, image acquisition was performed within a 4 h window on the 4th day of incubation (specifically 98–102 h of incubation). This time window was selected for two key reasons: (1) it falls within the early embryonic development stage before nociception formation, fully complying with animal welfare requirements; (2) chicken embryos at this stage have formed a complete vascular network, while the vessels have not yet been covered by the chorioallantoic membrane, ensuring clear transmission imaging of vascular features. Unfertilized eggs, dead embryos, and eggs accidentally damaged during acquisition were removed immediately. The remaining valid fertilized eggs were further incubated until hatching, and the true sex labels were determined by the fast–slow feathering method to construct a standard annotated dataset.

The main instruments and equipment used in this study included a fully automatic intelligent incubator, a darkroom transmission image acquisition device, an industrial camera, a 3 W LED green light, a computer (Intel Corporation, Santa Clara, CA, USA; NVIDIA Corporation, Santa Clara, CA, USA), an electronic balance (accuracy 0.01 g), a digital display vernier caliper (accuracy 0.01 mm), etc. The image acquisition device was independently designed to achieve stable light transmission and fixed-angle shooting, ensuring consistent acquisition conditions for all samples.

### 2.2. Image Acquisition System

**Figure 1:**
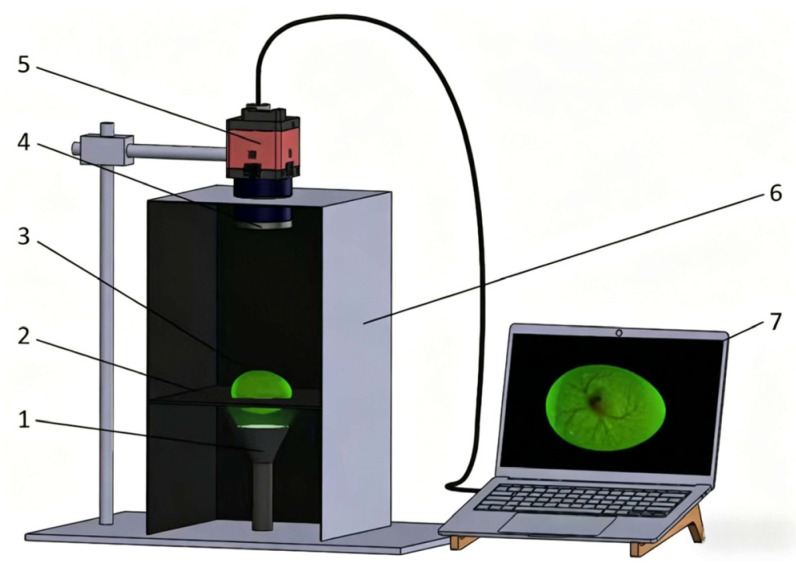
Image acquisition system for chicken embryo blood vessels. 1. Green LED light, 2. egg tray, 3. hatching eggs, 4. lens, 5. wide-angle camera, 6. dark box, and 7. computer.

To obtain clear, stable, and low-interference images of chicken embryo blood vessels, a darkroom transmission image acquisition system (Figure 1) was built in the testing laboratory of Shijiazhuang Yukou Poultry Industry Co., Ltd. The system consists of five parts: an industrial wide-angle camera, a transmission light source, a closed dark box, an egg fixing slot with an elliptical hole, and a computer. The dark box adopts an upper–lower split closed structure to effectively isolate external stray light interference; the camera is fixed in the upper cabin with the lens vertically downward, and the distance from the stage is fixed at 15 cm. The middle stage is fitted with a circular light-transmitting hole, above which an elliptical fixing slot is mounted to hold the hatching eggs horizontally and avoid the sample rolling or positional offset. The transmission light source is installed in the lower cabin with vertical upward irradiation and is kept 3 cm away from the stage to ensure uniform light penetration through the eggshell and clear visualization of embryonic blood vessels.

The industrial camera used was a Basler acA2040-180 km, with a resolution of 2048 × 2048 pixels, a global shutter, and a 16 mm fixed-focus lens(Basler AG, Ahrensburg, Germany). The image acquisition parameters were set as follows: an exposure time of 20 ms, a gain of 0 dB and an image output format of 8 bit grayscale. All acquisition parameters were fixed throughout the experiment to ensure consistent imaging conditions.

The LED transmission light source was optimized by an L9 (3^3^) orthogonal experiment, and the optimal parameters were determined as 3 W power and 520 nm wavelength green light. A 520 nm green light source was selected, as previous studies have confirmed that green light in this wavelength range has no adverse effect on the early development of chicken embryos and can significantly improve the contrast of blood vessels in transmission images [20]. During acquisition, hatching eggs were placed horizontally into the elliptical slot and left standing for 5 s to stabilize the embryonic posture before shooting. Images were stored in the computer in real time to ensure that all samples were collected under the same illumination, angle, and distance, minimizing the interference of environmental factors on the experimental results.

### 2.3. Sex True Label Annotation

On Day 19 of incubation, each hatching egg was numbered and placed in a sterile medical-grade non-woven fabric bag to ensure sample traceability. Sex identification was carried out within 24 h after hatching using the fast–slow feathering method [21]: under a cold LED light source, the absolute lengths of the primary wing feathers and primary coverts were measured with a digital display vernier caliper, and the difference was recorded. If the primary wing feathers were more than 2.00 mm longer than the primary coverts, the chick was judged as fast-feathering (female); otherwise, it was slow-feathering (male).

**Figure 2:**
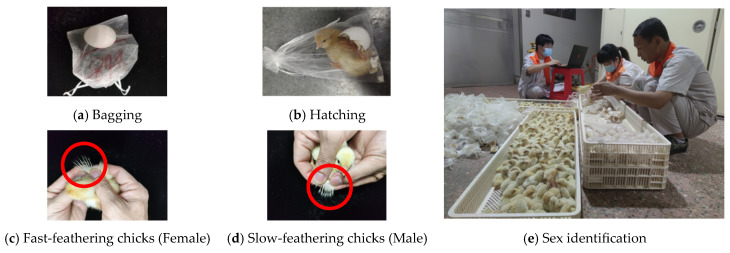
Chick sex verification.

The fast–slow feathering method is a widely recognized and genetically validated sex identification technique for Jingfen No. 1 layer chickens, with a reported accuracy of over 99% [22]. To ensure label accuracy, two senior hatchery technicians with more than 10 years of experience performed independent sex identification, and only consistent results were adopted as the final true sex labels. The verification process is shown in Figure 2.

### 2.4. Dataset Construction and Image Preprocessing

**Figure 3:**
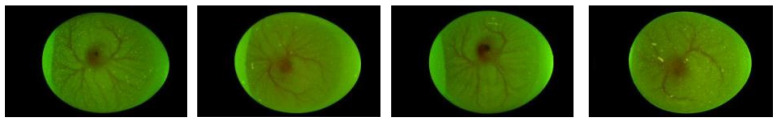
Blood vessel images of chicken embryos.

A total of 4011 valid images of chicken embryo blood vessels were collected on the 4th day of incubation (98–102 h) (Figure 3). All samples were further incubated until hatching and labeled with the true sex. Among them, 160 unhatched samples were excluded from model development and dataset partitioning, as their true sex labels could not be verified. The remaining 3851 valid hatched samples with confirmed sex labels included 1889 females and 1962 males. The female-to-male ratio was close to 1:1, which effectively avoids the impact of class imbalance on model training.

In accordance with the deep learning dataset partitioning standard, all valid samples were randomly divided into a training set (80%, 3081 images) and a held-out test set (20%, 770 images). The two sets were independently and identically distributed without repetition or crossover, ensuring the objectivity and reliability of model training and the test results. To further verify the stability of the model, 5-fold cross-validation was performed on the training set during model optimization, and the final model was evaluated on the independent held-out test set to avoid data leakage.

To improve the adaptability of the model under complex image conditions, online data augmentation was performed exclusively on the training set, with no augmentation applied to the test set. The augmentation methods included horizontal flips, vertical flips, random rotation (±15°), translation (±10%), Gaussian blur, random brightness adjustment (±15%), Gaussian noise, and random contrast variation. All augmentation operations did not destroy the basic structure of blood vessels and sex-related features, and the augmentation process was synchronized with training.

To unify the input size and enhance weak blood vessel features, standardized preprocessing was performed on all images: the original images were uniformly scaled to 224 × 224 pixels to fit the input requirements of the improved MobileViT-V3 model; limited adaptive contrast enhancement was applied to low-brightness and low-contrast images to highlight vascular textures; the egg region was located by ellipse fitting to remove the invalid black background outside the eggshell, which did not alter the vascular features within the egg region and introduced no bias to feature extraction; the image pixel values were normalized to the interval [0, 1] to accelerate model’s convergence and improve training stability.

## 3. Establishment of the Improved MobileViT-V3 Model

### 3.1. Network Architecture of MobileViT-V3

MobileViT-V3 is a lightweight convolutional-Transformer hybrid architecture that combines the local detail extraction capability of CNN and the global modeling advantage of Transformer. It enables efficient and stable visual feature learning on low-computation platforms. The network consists of downsampling layers, multi-level feature extraction modules, and a global pooling classification layer. It extracts multi-scale semantic information through hierarchical downsampling and lightweight Transformer modules, delivering strong feature representation while ensuring inference speed. However, the standard MobileViT-V3 has limited perception of tiny, low-contrast objects and is prone to missing or confusing thin, weak-signal features such as chicken embryo blood vessels. Therefore, this study takes MobileViT-V3 as the base network and performs lightweight improvements tailored to the thin, weak, and easily interfered characteristics of blood vessels so as to enhance the model’s capability in capturing tiny targets and its anti-interference performance.

### 3.2. Overall Network Architecture of the Improved MobileViT-V3

In response to the biological characteristics of chicken embryo blood vessels on the fourth day of incubation—weak, thin, easily submerged by noise, and direction-sensitive—this study made targeted improvements to MobileViT-V3 to construct a closed-loop feature extraction mechanism of local enhancement–global screening. The overall improvement strategy is as follows: embed the Micro Feature Enhancement module (MFE-Module) after patch embedding in Stage 3 to strengthen the details of tiny blood vessels and low-contrast features; embed the Multi-Scale Adaptive Attention Fusion module (MSAAF-Module) after Stage 4 to realize adaptive weighted screening of multi-source features. The specific improvement ideas are as described below.

Micro Feature Enhancement module (MFE-Module): This performs focused enhancement on weak blood vessels through local blocking, linear encoding–decoding, learnable prompt vectors, sparse masks, and CBAM spatial attention [23], solving the problem that traditional models struggle to capture tiny blood vessels. This module compresses redundant information while retaining and strengthening highly discriminative local details and preserves low-level image information via residual connections to avoid gradient vanishing.

Multi-Scale Adaptive Attention Fusion module (MSAAF-Module): This adopts a three-branch parallel structure, extracting the directional features of blood vessels via asymmetric convolutions, capturing key regions via CBAM spatial attention, and enhancing effective channels via SENet-like channel attention. Then it realizes adaptive weighted fusion through the learnable weights α, β, and γ to automatically screen the most effective features for gender classification.

**Figure 4:**
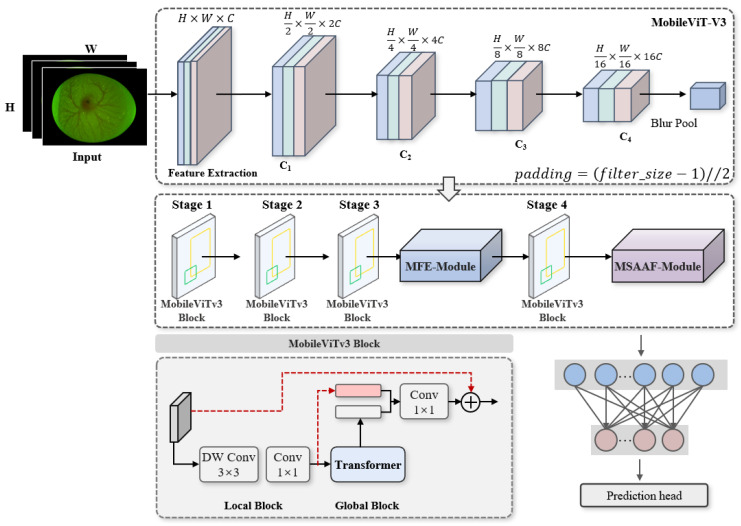
Structure of the improved MobileViT-V3 model.

Collaborative synergy of two modules: the MFE-Module strengthens local details in shallow layers, and the MSAAF-Module completes global screening and feature fusion in deep layers, forming a robust pipeline of “enhancement first, screening later”. This significantly improves the classification accuracy and generalization ability of the model under low-quality images (overexposure, overdarkness, bubble occlusion, blurring). The structure of the improved MobileViT-V3 model is shown in Figure 4.

### 3.3. Micro Feature Enhancement Module (MFE-Module)

The self-attention mechanism of traditional ViT focuses more on global semantics and tends to lose fine local features, while standard convolutions lack content adaptability and cannot accurately extract weak blood vessels. To address these issues, this study designed the MFE-Module to perform focused feature enhancement at the regional level, specially adapted to the thin, weak, and locally concentrated characteristics of chicken embryo blood vessels.

Different from traditional vision transformers that directly conduct global encoding on patches, the MFE-Module first compresses features along the channel dimension to highlight spatial morphological information, which is more suitable for spatial topological features such as blood vessels. Given an input feature tensor $X\in R^{B\times C\times H\times W}$ (where B is the batch size, C is the channel number, H is the height, and W is the width), the patch size P=2 is set to split the feature map into non-overlapping patch tensors $X\in R^{B\times N\times D_{p}\times C}$, where $N=\left(\frac{H}{P}\right)\times \left(\frac{W}{P}\right)$ is the total number of patches, and $D_{p}=4$ is the spatial dimension of a single flattened patch.

The mean value is taken along the channel dimension to eliminate redundant information between channels, obtaining a pure spatial region descriptor that highlights the spatial structure of blood vessels. Since this descriptor has an extremely low dimension and cannot be directly used for semantic modeling, a linear encoder + linear decoder structure is adopted to complete nonlinear mapping: the encoder compresses low-dimensional spatial features into a latent semantic space to extract abstract features; the decoder restores features to the original channel dimension to convert spatial information into high-dimensional semantic features. The encoding and decoding formula is shown in Equation (1)

(1) $$Y_{decoded}=LayerNorm\left(X_{raw}\cdot W_{e}^{T}+b_{e}\right)\cdot W_{d}^{T}+b_{d}$$

where $X_{raw}$ is the patch spatial feature tensor; $W_{e}$ and $W_{d}$ are learnable weight matrices for encoding and decoding, respectively; $b_{e}$ and $b_{d}$ are corresponding biases; LayerNorm denotes layer normalization; and $Y_{decoded}$ is the decoded output feature.

**Figure 5:**
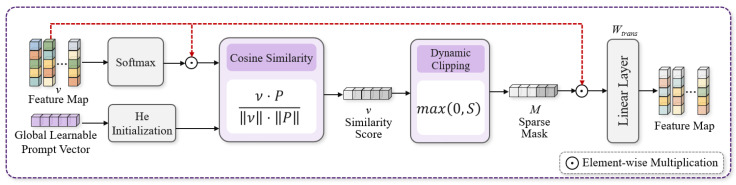
Global learnable vector module.

After encoding and decoding, different regional features contribute differently to gender classification, and regions such as eggshells, air cells, and bubbles cause interference. To this end, a global learnable prompt vector $P\in R^{C}$ is introduced, initialized with He initialization and updated synchronously with the network to guide the model to focus on blood vessel-related regions. Its architecture is shown in Figure 5.

First, Softmax normalization enhancement is performed on the local features

(2) $$v_{i}^{\prime }=v_{i}⊙Softmax\left(v_{i}\right)$$

where $v_{i}$ is the i-th regional feature, ⊙ denotes element-wise multiplication, and ${v_{i}}^{\prime }$ is the enhanced feature.

The cosine similarity between the enhanced feature and the prompt vector is calculated to measure the relevance between the region and the task target

(3) $$S_{i}=\frac{V_{i}\prime \cdot P}{\left\|V_{i}\prime \right\|\cdot \left\|P\right\|}$$

where P is the global learnable prompt vector and $S_{i}$ is the similarity score.

A sparse mask is generated through dynamic truncation to filter low-relevance regions and retain high-response regions

(4) $$M_{i}=max\left(0,S_{i}\right)\;m_{i}={v_{i}}^{\prime }⊙M_{i}$$

Finally, feature reconstruction is completed via the learnable transformation matrix $W_{trans}\in R^{C\times C}$

(5) $$f_{i}=m_{i}\cdot W_{trans}$$

This pipeline automatically suppresses background noise, accurately enhances blood vessel regions, and significantly improves the distinguish ability of weak features.

To further precisely focus on blood vessel regions, the CBAM spatial attention module is introduced. Channel average pooling and max pooling are performed in parallel on the reconstructed features to fuse global statistical information and local salient information; a spatial weight mask is generated through 3×3 convolution and Sigmoid activation to realize adaptive enhancement of target regions.

**Figure 6:**
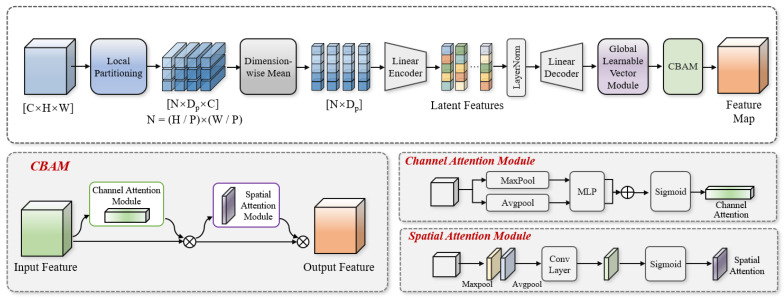
Micro Feature Enhancement Module (MFE-Module).

Ultimately, the enhanced features and input features are connected via residual connection, which not only alleviates the gradient vanishing problem of deep networks but also preserves low-level image details, realizing multi-scale fusion of high-level semantics and low-level spatial features, and making tiny blood vessel features more prominent and stable. The overall architecture of the MFE-Module is shown in Figure 6.

### 3.4. Multi-Scale Adaptive Attention Fusion Module (MSAAF-Module)

After enhancement by the MFE-Module, the features have clearer local details but still suffer from problems such as multi-scale redundancy, disordered directional information, and uneven channel contributions. Therefore, the MSAAF-Module was designed to adopt a three-branch parallel structure of asymmetric convolutions + spatial attention + channel attention, realizing adaptive weighted fusion of multi-source features and finally outputting highly discriminative global features.

**Figure 7:**
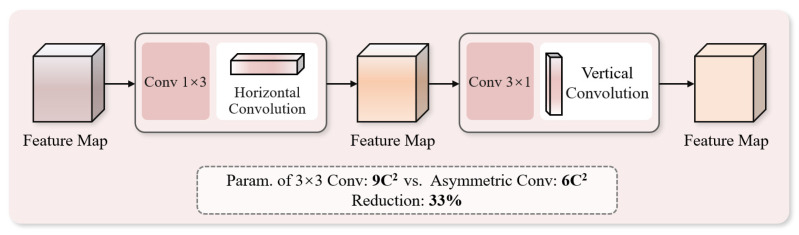
Asymmetric convolution module.

Chicken embryo blood vessels present a slender network topology with obvious horizontal and vertical directionality, which standard 3 × 3 isotropic convolutions cannot efficiently capture. This study splits standard convolutions into 1 × 3 and 3 × 1 asymmetric convolutions to extract the horizontal and vertical structural features, respectively. For C channels, the parameter amount of standard convolution is 9C^2^, while that of asymmetric convolution is only 6C^2^, reducing the computational cost by 33% and significantly improving efficiency. This structure forces the model to extract features along the horizontal and vertical axes, making it more sensitive to slender blood vessel networks and effectively capturing differences in blood vessel trends. Its architecture is shown in Figure 7.

The MSAAF-Module adopts a three-branch parallel structure to achieve omni-directional feature extraction: capturing the horizontal and vertical directional features of blood vessels via asymmetric convolutions, and completing feature recalibration through 1 × 1 convolution and Sigmoid activation to highlight linear structures; adopting CBAM spatial attention to generate spatial weight maps through dual pooling and convolution, automatically focusing on blood vessel regions and suppressing interference such as eggshells, bubbles, and shadows; and adopting SENet-like channel attention to compress spatial information through global average pooling, learning channel importance via two fully connected layers, and strengthening feature channels related to gender. The three branches complement each other and complete feature screening according to direction, spatial position, and channel importance, fully covering blood vessel discrimination information.

To enable the model to automatically select the optimal feature combination based on data, the learnable weight parameters α, β, and γ are introduced with an initial value of 0.1 to ensure balanced contribution of the three branches in the early training stage, and they are adaptively optimized during training. The three-branch outputs are weighted and fused

$$F_{\mathrm{fusion}}=\alpha \cdot F_{\mathrm{direction}}+\beta \cdot F_{\mathrm{space}}+\gamma \cdot F_{\mathrm{channel}}$$

**Figure 8:**
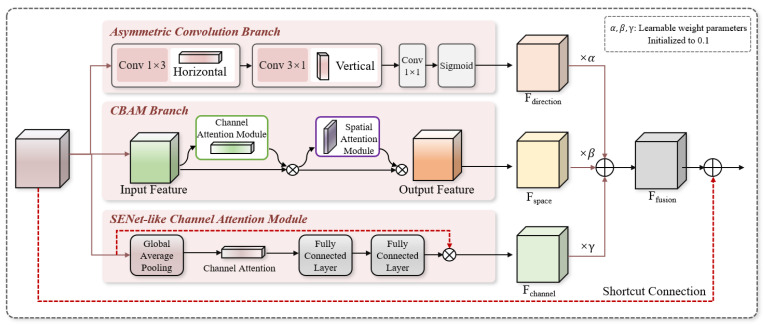
Multi-Scale Adaptive Attention Fusion Module (MSAAF-Module).

Finally, the fused features and original input features are connected via residual connection to achieve information enhancement and stable gradient propagation. This mechanism enables the model to stably output highly discriminative features even when facing complex images such as blurring, overdarkness, offset, and bubble occlusion, greatly improving robustness and generalization ability. The overall architecture of the MSAAF-Module is shown in Figure 8.

## 4. Results and Analysis

### 4.1. Evaluation Indicators

In this study, five standard and widely accepted metrics were adopted to comprehensively evaluate the model’s classification performance, including accuracy, precision, recall, F1-score, and Kappa coefficient. The calculation formulas of all indicators follow the standard definitions in previous studies, as shown below

$$Accuracy=\frac{TP+TN}{TP+TN+FP+FN}$$

where TP (true positive) is the number of female embryos correctly predicted as female, TN (true negative) is the number of male embryos correctly predicted as male, FP (false positive) is the number of male embryos incorrectly predicted as female, and FN (false negative) is the number of female embryos incorrectly predicted as male.

### 4.2. Model Comparison Experiments

To comprehensively verify the effectiveness of the improved MobileViT-V3, it was quantitatively compared with current mainstream visual classification models, including YOLOv12 [24], ShuffleNetV2 [25], ConvNeXt-T [26], ResNet [27], ViT [28], Swin-ViT [29], and the original MobileViT-V3.

All comparison models were trained and tested using identical hardware (Intel Core i9 processor, NVIDIA A800 GPU), software (PyTorch 2.0 framework), and training strategies: 100 training epochs, AdamW optimizer with an initial learning rate of 1 × 10^−4^, a batch size of 32, and cosine learning rate decay. All models were initialized with pre-trained weights on ImageNet-1K to ensure a fair comparison.

**Figure 9:**
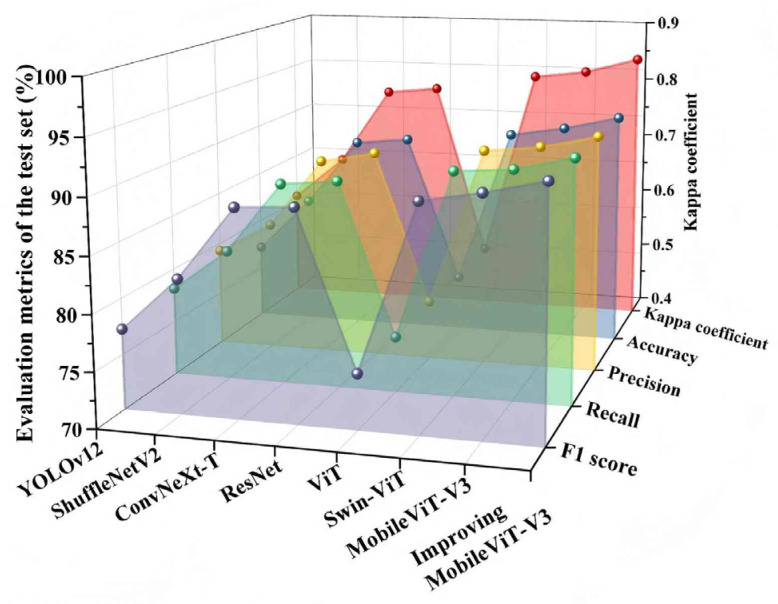
Performance comparison of mainstream models.

The experimental results show that different network architectures exhibit significant performance differences in the high-noise, low-contrast, and fine-grained classification task of weak blood vessels of chicken embryos on the fourth day of incubation, as shown in Figure 9. YOLOv12, which is primarily optimized for target detection tasks, showed limited performance in fine-grained weak texture feature extraction for this classification task, achieving an accuracy of 77.14%. ShuffleNetV2, an extremely lightweight convolutional model, had limited feature expression capability, with an accuracy of 82.34%. ConvNeXt-T and ResNet achieved accuracies of 88.72% and 89.25%, respectively, as their pure convolutional structures lacked long-range global dependency modeling capability, leading to the dilution of scattered slender vascular features during continuous downsampling. The native ViT had a global receptive field but lacked spatial inductive bias, resulting in poor perception of tiny vascular details and an accuracy of only 75.27%. Swin-ViT improved local perception through a window mechanism, achieving an accuracy of 90.17%, but its fixed window limited global information interaction for vascular network features. The original MobileViT-V3 achieved an accuracy of 90.87%, striking a certain balance between lightweight and accuracy, but there was still room for improvement in weak feature extraction.

The improved MobileViT-V3 proposed in this paper achieves a test set classification accuracy of 92.26% through micro feature enhancement and multi-scale adaptive attention fusion, with a precision of 92.18%, a recall of 92.12%, an F1-score of 92.15%, and a Kappa coefficient of 0.845. Its comprehensive performance is superior to all comparison models. The improved model achieved a 1.39 percentage point improvement in accuracy over the original MobileViT-V3. Although the absolute improvement is modest, it is meaningful for industrial applications, as even a 1% increase in accuracy can reduce the number of misclassified eggs by tens of thousands in large-scale hatcheries with millions of hatching eggs processed daily.

### 4.3. Ablation Experiment Results of the Improved MobileViT-V3

To verify the effectiveness of each improved module, ablation experiments were carried out with the original MobileViT-V3 as the baseline (Table 1). The experimental results show that the accuracy of the baseline MobileViT-V3 is 90.87%. After adding the MSAAF-Module only, the accuracy increases to 91.68% and the recall increases to 92.03%, indicating that multi-branch attention and asymmetric convolutions can effectively screen direction, spatial, and channel features, reducing the missed detection rate. After adding the MFE-Module only, the accuracy increases to 91.84%, indicating that this module can significantly enhance the details of weak blood vessels and improve features’ distinguishability.

When both modules were added, the model achieved the optimal performance, with an accuracy of 92.26%, 1.39 percentage points higher than the baseline. The combined improvement from both modules exceeded the sum of individual improvements (0.81% from the MSAAF-Module alone and 0.97% from the MFE-Module alone), indicating a positive synergistic effect between the two modules. Specifically, the MFE-Module enhanced local micro-vascular features in shallow layers, while the MSAAF-Module completed global feature screening and fusion in deep layers, forming a complementary feature extraction pipeline. The F1-score and recall also increased synchronously, with the Kappa coefficient increasing to 0.845. The results prove the necessity and effectiveness of the improvement scheme.

In terms of model complexity, the number of parameters of the improved model increases from 2.74 M to 2.98 M, with an increase of about 8.7%; the computational FLOPs increase from 558.32 M to 568.40 M, with an increase of about 1.8%; and the inference speed decreases from 102.3 FPS to 97.6 FPS. The real-time requirement of industrial sorting lines is usually ≥30 FPS, and the speed of the model in this paper is much higher than the industrial standard, achieving a favorable balance between classification accuracy and computational efficiency.

### 4.4. Visualization Analysis

To comprehensively evaluate the classification effect, feature focusing ability, and practical engineering applicability of the improved MobileViT-V3 model in the early non-destructive sex identification of chicken embryos, this study carried out a visualization analysis from four dimensions, namely classification consistency, feature response distribution, global threshold robustness, and positive class discrimination reliability, to intuitively verify the extraction effect and anti-interference ability of the model on weak blood vessel features.

**Figure 10:**
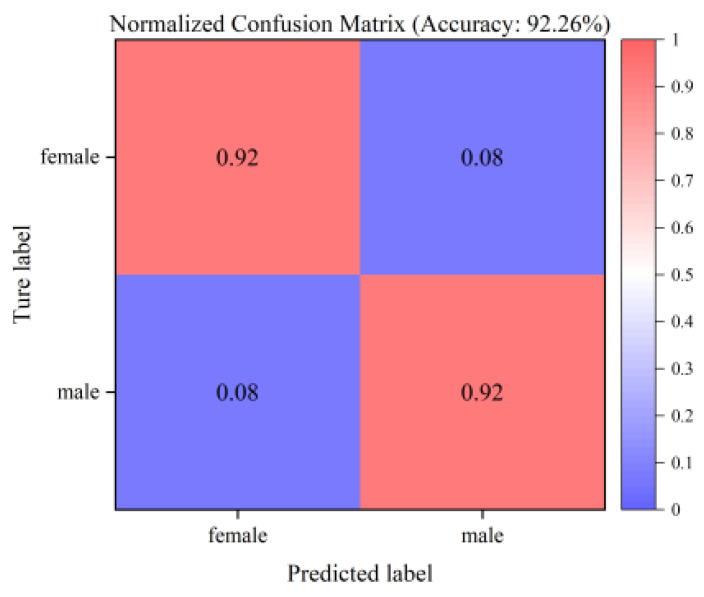
Normalized confusion matrix.

The results of the normalized confusion matrix (Figure 10) show that the identification accuracies of the improved MobileViT-V3 model proposed in this paper for female and male chicken embryos are 92.31% and 92.20%, respectively, with excellent overall classification balance and no obvious class bias. To quantify the model’s performance under non-ideal conditions, we further divided the test set into four subsets based on image quality: normal images (420 samples), low-brightness/overexposed images (120 samples), bubble-occluded images (130 samples), and blurry images (100 samples). The model achieved accuracies of 94.05%, 90.83%, 90.00%, and 89.00% on these subsets, respectively, demonstrating stable performance even under challenging non-ideal imaging conditions, with both false positive and false negative rates controlled at a low level. This indicates that the model can effectively suppress interference such as eggshell texture, air chamber, and illumination noise; accurately capture the subtle differences between the blood vessels of female and male chicken embryos; and achieve stable and reliable fine-grained classification.

**Figure 11:**
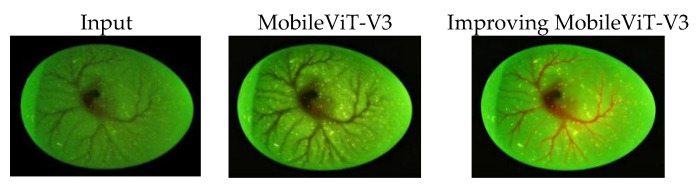
Visualization of feature response.

The results of feature response visualization [30] (Figure 11) show that the improved model can automatically focus on the distribution area of chicken embryo blood vessels, generating significant responses to tiny blood vessels, bifurcation points, and trend features, while the activation values for eggshells, backgrounds, and invalid areas are low. To quantitatively compare the feature focusing ability of the original and improved models, we calculated the average activation value within the manually labeled vascular region and the background region for all test set samples. The improved model achieved a vascular-to-background activation ratio of 7.82, significantly higher than the 4.35 of the original MobileViT-V3, quantitatively confirming that the improved model can more effectively focus on vascular regions and suppress background interference. Compared with the original MobileViT-V3, the heatmap of the improved model is more concentrated and the boundaries are clearer, which can accurately locate the key feature areas highly related to gender. This proves that the Micro Feature Enhancement Module can effectively strengthen weak blood vessel signals, and the Multi-Scale Adaptive Attention Fusion Module can screen highly discriminative features, achieving the ideal effect of focusing on key areas and suppressing redundant information.

**Figure 12:**
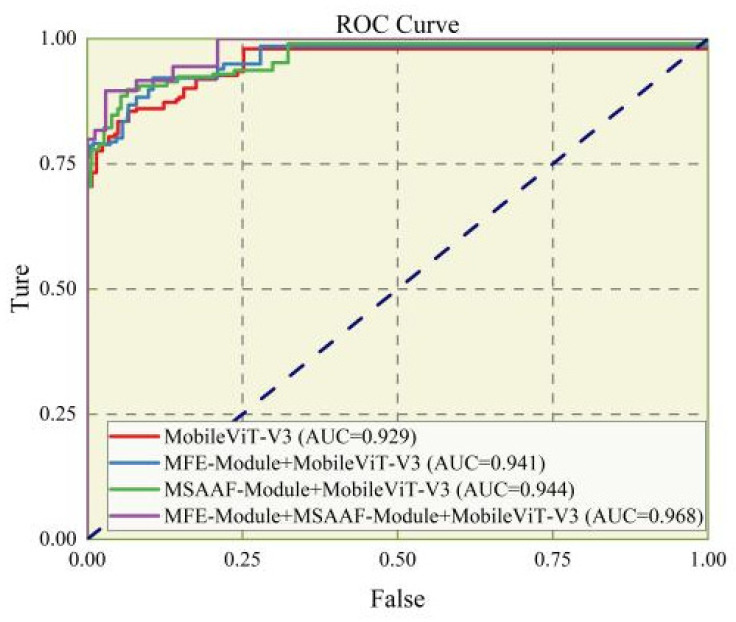
ROC curve of the improved model.

**Figure 13:**
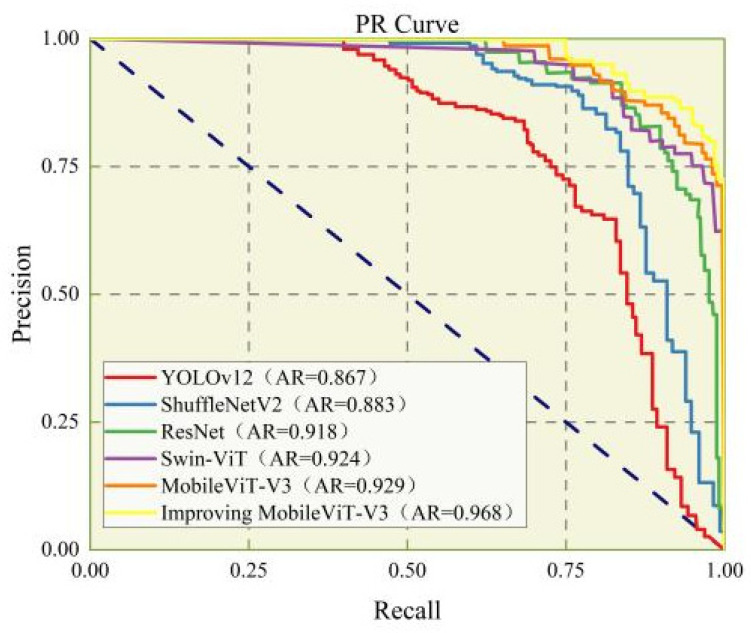
PR curves of different models.

The results of the ROC curve [31] (Figure 12) show that the ROC curve of the improved MobileViT-V3 is generally close to the upper left corner, with an area under the curve (AUC) of 0.968, indicating that the model can still maintain a very high true positive rate under extremely low false positive rate conditions, with excellent cross-threshold robustness. Under different sensitivity requirements of industrial sorting, the model can maintain stable identification performance, which can flexibly adapt to the actual production requirements on site; the PR curve (Figure 13) further verifies the reliability of the model in actual production. The PR curve shows that the model maintained a precision above 0.95 when recall reached 0.9, indicating a low false positive rate. This characteristic is highly consistent with the core requirement of the hatching industry to minimize the misclassification of female embryos as male, as such misclassification would lead to direct economic losses for hatcheries. Before the recall reaches 0.9, the precision of the improved model is basically maintained around 1.0, with an average precision (AP) value of 0.968, which is significantly better than the other comparison models.

Comprehensive analysis of the visualization results above demonstrates that the improved MobileViT-V3 can effectively extract the weak vascular features of early chicken embryos via local micro feature enhancement and global multi-scale attention screening. Meanwhile, the model can suppress complex background interference and achieve a favorable balance among classification accuracy, class balance, and model robustness.

## 5. Discussion

The global poultry industry has an urgent demand for early, non-destructive in ovo sex identification technologies compliant with animal welfare to replace the routine culling of day-old male chicks banned in multiple countries. Existing methods either fail to achieve high-accuracy identification of 4-day-incubated embryos or cannot balance detection accuracy and lightweight industrial deployment, limiting their large-scale application. This study developed an improved MobileViT-V3-based method for early non-destructive sex identification of chicken embryos, effectively enhancing the model’s perception of weak vascular features and industrial adaptability.

Compared with existing studies focusing on the mid–late incubation stages or large-parameter models, this method achieves 92.26% identification accuracy on 4-day-incubated embryo samples, outperforming the mainstream classification models. With only 2.98 M parameters and a 97.6 FPS inference speed, it effectively balances high-precision identification and high-throughput industrial deployment requirements, providing a feasible solution for early online embryo detection. The lightweight module design also offers a reference for similar weak target fine-grained classification tasks.

This study has two main limitations. First, validation is limited to Jingfen No. 1 white-shell layer eggs, so the model’s generalizability to other breeds or eggshell colors needs further verification. Second, the model was only tested under laboratory conditions, and its stability in complex industrial scenarios requires on-site pilot validation.

Overall, the proposed method fully meets animal welfare requirements, with the advantages of low cost, easy deployment, and high throughput. It can support the poultry hatching industry’s transformation away from male chick culling, and can promote the green development of poultry breeding.

## 6. Conclusions and Outlook

This study addresses the core bottleneck of low identification accuracy for weak vascular features in 4-day-incubated chicken embryos and develops a lightweight non-destructive visual method for early embryo sex identification. The key finding is that the proposed method achieves 92.26% identification accuracy on the self-constructed dataset, with real-time inference performance fully meeting industrial high-speed sorting requirements.

The core practical contribution of this work is to provide a low-cost, scalable early in ovo sex identification scheme compliant with global animal welfare regulations. This scheme effectively alleviates the resource waste and animal ethical issues caused by day-old male chick culling and has clear industrial application value.

Future research will focus on three directions: first, building a multi-breed, multi-scenario dataset to improve the model’s generalizability; second, completing embedded deployment and industrial line pilot testing of the model; and third, exploring multi-modal fusion to further enhance identification stability.

## Figures and Tables

**Table 1:** Comparison of the ablation experiment’s results.

MFE-Module	MSAAF-Module	Accuracy (%)	Precision (%)	Recall (%)	F1-Score (%)	Kappa Coefficient
×	×	90.87	90.82	90.75	90.79	0.819
×	√	91.68	91.59	92.03	91.81	0.833
√	×	91.84	91.79	91.72	91.75	0.837
√	√	92.26	92.18	92.12	92.15	0.845

Note: “√” indicates that the corresponding module is added to the model, “×” indicates that the corresponding module is not added to the model.

## Data Availability

The data presented in this study are available within the article. The original contributions presented in this study are included in the article. Further inquiries can be directed to the first author (Qian Yan, yqyq@webmail.hzau.edu.cn).
